# Supplementary material for: Impact of Green Chitosan Nanoparticles Fabricated from Shrimp Processing Waste as a Source of Nano Nitrogen Fertilizers on the Yield Quantity and Quality of Wheat (Triticum aestivum L.) Cultivars
Source: Molecules. 2022 Sep 1;27(17):5640. doi: 10.3390/molecules27175640 (PMC9457985; doi:10.3390/molecules27175640)
Supplement: Supplementary file 1 [file molecules-27-05640-s001.zip › molecules-1896372-supplementary.pdf]

Supplementary data

I. Figures

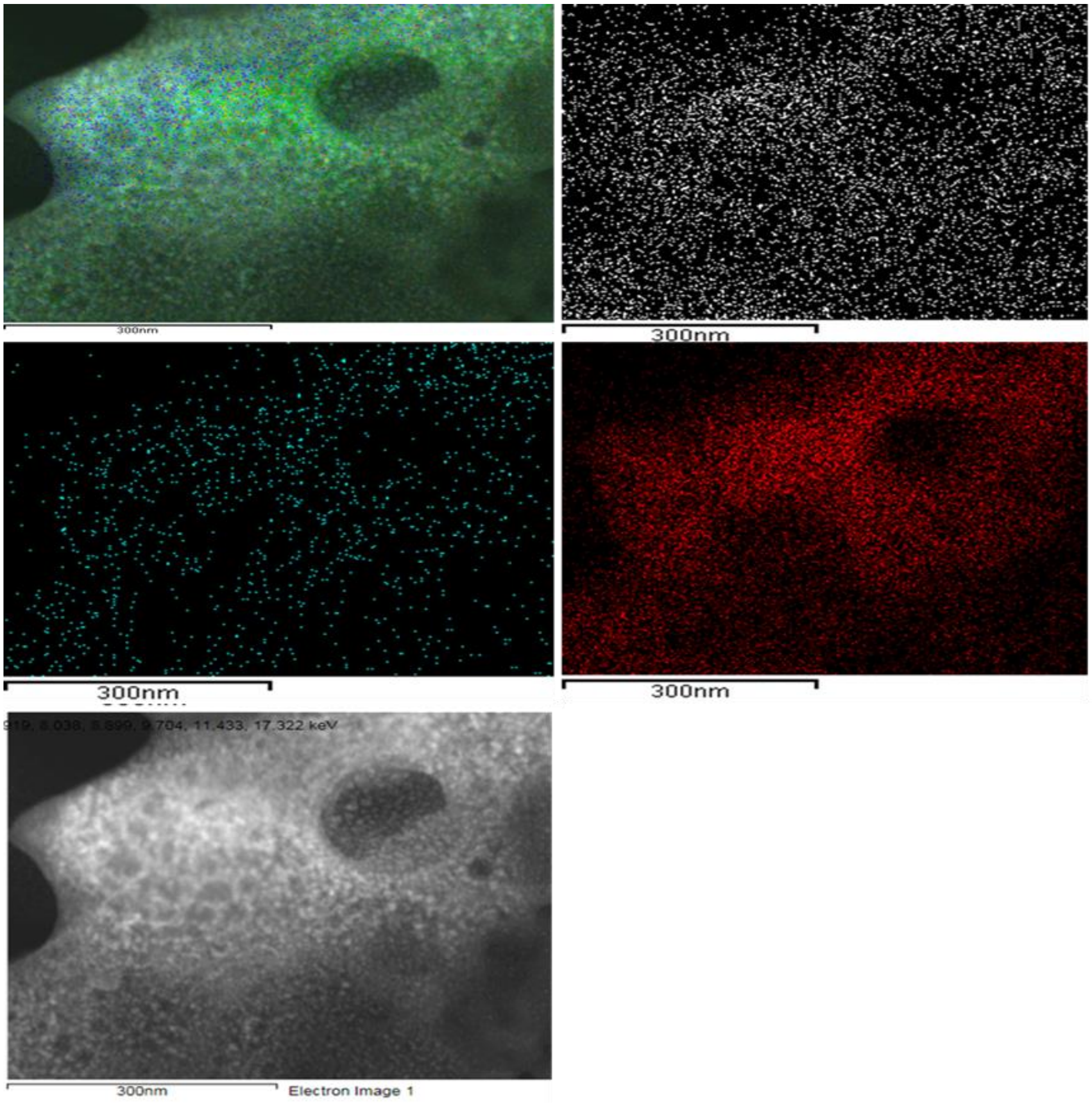

Figure S1. Electron image of the energy dispersive X-ray (EDX) for chitosan nanoparticles.

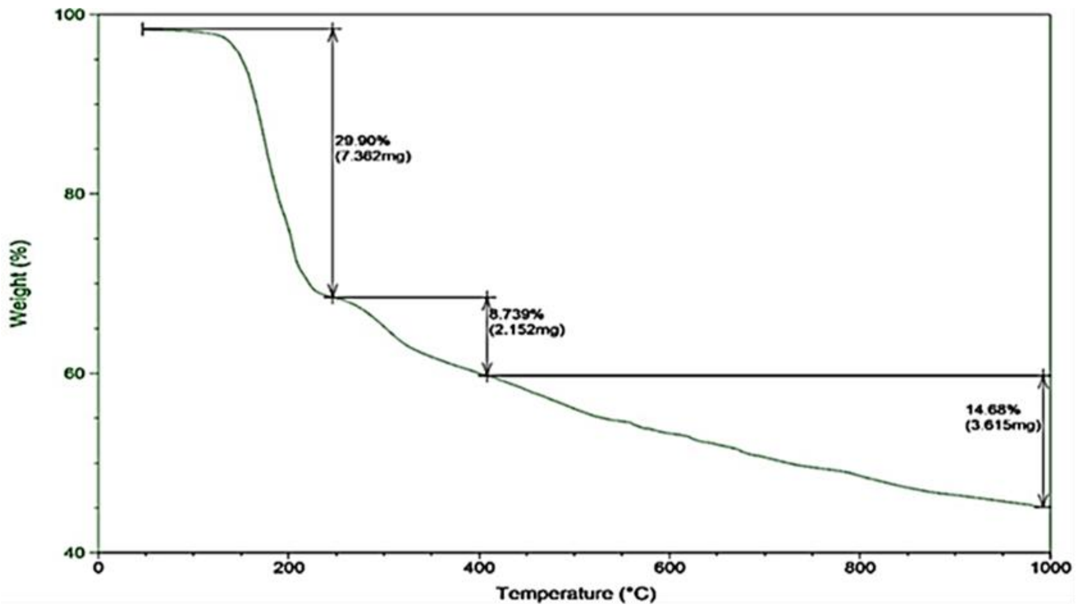

Figure S2. TGA of chitosan nanoparticles.

II. Tables

Supplementary Table S1. Physical and chemical properties of the experimental soil units during the growing seasons 2019/2020, 2020-2021.

Properties	First season	First season
**Physical analysis:**		
Course sand %	5.90	4.82
Find sand %	19.60	8.05
Silt %	28.20	36.60
Clay %	46.30	50.53
Texture grade	Clay	Clay
**Chemical analysis:**		
pH (1: 2.5)	7.8	8.0
E.C. (ds/m) (1:20)	0.16	0.22
$CaCO_3$ (%)	3.14	2.13
HCO_3 (meq/L)	1.25	1.25
Cl^- (meq/L)	0.58	0.55
Ca^{++} (meq/L)	0.8	0.9
Na^+ (meq/L)	0.77	0.83
K^+ (meq/L)	0.24	0.18
Mg^{++} (meq/L)	0.2	0.2
N available (mg/kg)	247	172
P available (mg/kg)	6.0	12
K available (mg/kg)	1360	1270

Table S2. Impact of nano and mineral nitrogen fertilizers on straw yield kg/ha of two wheat cultivars during 2019/2020 and 2020/2021 seasons.

Fertilization Source (F)	First Season			Second Season		
	Cultivar (C)		Mean (F)	Cultivar (C)		Mean (F)
	Gemaiza-11	Misr-1		Gemaiza-11	Misr-1	
Untreat-control (without N) Mn-	9587±312	10565±395	**10076±387** ^{F}	11976±380 ^{f}	12800±322 ^{ef}	**12388±375** ^{D}
N (120 kg /ha)	15251±356	13753±364	**14502±349** ^{DE}	12812±323 ^{ef}	14787±436 ^{cdef}	**13800±382** ^{CD}
Mn-N (240 kg /ha)	15544±491	15086±342	**15315±303** ^{CD}	12844±306 ^{ef}	13524±311 ^{def}	**13184±366** ^{D}
Nan-N (7L/ha)	13778±428	13537±289	**13657±259** ^{E}	15867±339 ^{abcde}	15315±358 ^{bcde}	**15591±354** ^{BC}
Nan-N (14L/ha)	13817±325	13309±355	**13563±369** ^{E}	16267±343 ^{abcd}	15683±367 ^{abcde}	**15975±323** ^{BC}
Mn-N 120kg/ha + Nan-N 7L/ha	17499±366	15455±398	**16477±343** ^{C}	15849±278 ^{abcde}	13906±391 ^{cdef}	**14878±304** ^{C}
Mn-N 120kg/ha + Nan-N 14L/ha	18464±306	17145±412	**17804±429** ^{B}	17893±352 ^{abc}	16858±382 ^{abc}	**17375±415** ^{AB}
Mn-N 240kg/ha + Nan-N 7L/ha	19937±318	18045±457	**18991±416** ^{B}	18401±424 ^{ab}	18921±437 ^{a}	**18661±374** ^{A}
Mn-N 240kg/ha + Nan-N 14L/ha	22032±345	19442±377	**20737±308** ^{A}	18585±396 ^{ab}	18261±454 ^{ab}	**18423±425** ^{A}
**Mean (C)**	**16212±307** ^{A}	**15148±324** ^{B}		**15611±327**	**15562±342**	

ANOVA	df	First Season	Second Season
Cultivar (C)	1	0.041	0.806

Fertilization Source	8	<0.001	<0.001
(F) F×C	8	0.450	0.049

Different uppercase letter indicates significant difference among evaluated cultivars or fertilization source at $p < 0.05$, while different lowercase letter indicates significant difference among their interaction.
